# The Role of Lytic Infection for Lymphomagenesis of Human γ-Herpesviruses

**DOI:** 10.3389/fcimb.2021.605258

**Published:** 2021-03-26

**Authors:** Christian Münz

**Affiliations:** Viral Immunobiology, Institute of Experimental Immunology, University of Zürich, Zürich, Switzerland

**Keywords:** Epstein Barr virus, Kaposi sarcoma associated herpesvirus, viral IL-10, viral IL-6, viral G-protein coupled receptor, early lytic EBV antigen specific T cells, natural killer cells

## Abstract

Epstein Barr virus (EBV) and Kaposi sarcoma associated herpesvirus (KSHV) are two oncogenic human γ-herpesviruses that are each associated with 1-2% of human tumors. They encode bona fide oncogenes that they express during latent infection to amplify their host cells and themselves within these. In contrast, lytic virus particle producing infection has been considered to destroy host cells and might be even induced to therapeutically eliminate EBV and KSHV associated tumors. However, it has become apparent in recent years that early lytic replication supports tumorigenesis by these two human oncogenic viruses. This review will discuss the evidence for this paradigm change and how lytic gene products might condition the microenvironment to facilitate EBV and KSHV associated tumorigenesis.

## Introduction on EBV and KSHV Infection and Tumorigenesis

Epstein Barr virus (EBV) and Kaposi sarcoma associated herpesvirus (KSHV) are the two human γ-herpesviruses (human herpesvirus 4 and 8, respectively) (Ehlers et al., 2010). Both are quite successful pathogens in the human population and have no other known animal hosts (Münz, 2019; Cesarman et al., 2019). EBV persistently infects more than 95% of the human adult population, and while rare in the Northern hemisphere, persistent KSHV infection is found in more than 50% of individuals in sub-Saharan Africa. Both viruses encode latent and lytic gene products (Mariggio et al., 2017; Frost and Gewurz, 2018). While lytic replication allows through the expression of immediate early, early and late structural γ-herpesvirus genes the production of infectious viral particles, latent gene expression is thought to maintain viral DNA in proliferating lymphocytes, probably primarily B cells, rescue them from apoptosis and drive them into differentiation to long-lived memory cells for persistence (Thorley-Lawson, 2001; Dittmer and Damania, 2016; Frohlich and Grundhoff, 2020). For this purpose, EBV encodes six latent nuclear (EBNA1, 2, 3A, 3B, 3C and LP), two latent membrane (LMP1 and 2) and non-translated RNAs (Epstein–Barr virus-encoded small RNAs or EBERs and miRNAs) (Kempkes and Robertson, 2015). They are grouped in four latency patterns (0, I, II and III). Latency III expresses all latency genes and can be found in naïve B cells of healthy virus carriers, latency II only EBNA1, LMP1 and 2 plus non-translated RNAs in germinal center B cells, latency I only EBNA1 at the protein level in homeostatically proliferating memory B cells and latency 0 only non-translated RNAs in resting memory B cells as the site of EBV persistence (Babcock et al., 2000; Hochberg et al., 2004). KSHV expresses latency-associated nuclear antigen (LANA), vCyclin, viral FADD-like interleukin-1-beta-converting enzyme [FLICE/caspase 8]-inhibitory protein (vFlip), Kaposins and non-translated RNAs (miRNAs) during latency (Frohlich and Grundhoff, 2020). However, lytic KSHV gene products are often co-expressed even in poorly infectious virus-producing cells, and KSHV latency has not yet been linked to a human B cell differentiation program.

Nevertheless, latent EBV and KSHV gene products have oncogenic abilities and, therefore, both human γ-herpesviruses are designated WHO class I carcinogens (Parkin, 2006; Bouvard et al., 2009). Although EBV encodes the more potent growth transforming gene products (Kulwichit et al., 1998; Sin and Dittmer, 2013; Sin et al., 2015; AlQarni et al., 2018) and can readily immortalize human B cells *in vitro* (Pich et al., 2019), each of the two viruses is associated with around 1-2% of all cancers in humans (Cesarman, 2014; Cesarman et al., 2019; Shannon-Lowe and Rickinson, 2019). These include primarily B cell lymphomas and epithelial cell carcinomas for EBV, and endothelial cell cancers and B cell lymphoproliferations for KSHV. EBV latency I is present in Burkitt’s lymphoma, latency II in Hodgkin’s lymphoma and nasopharyngeal carcinoma, and latency III in some diffuse large B cell lymphomas (DLBCL) and post-transplant lymphoproliferative disease (PTLD) (Shannon-Lowe and Rickinson, 2019). KSHV is associated with the endothelial cell tumor Kaposi sarcoma and multicentric Castleman’s disease (Cesarman, 2014; Cesarman et al., 2019). Finally, primary effusion lymphoma (PEL), a plasmacytoma (Klein et al., 2003), is to 100% associated with KSHV and harbors in addition EBV in 90% of cases (Cesarman et al., 1995; Nador et al., 1996; Cesarman, 2011). Interestingly, it is also the only KSHV associated tumor, from which readily transformed cell lines can be established *in vitro* that maintain KSHV (Frohlich and Grundhoff, 2020). Interestingly, co-infection with EBV allows KSHV persistence in mice with reconstituted human immune system components (humanized mice), and results in PEL-like lymphomagenesis (McHugh et al., 2017). Similarly, the two γ-herpesviruses or their monkey orthologues seem to be also co-transmitted in Cameroonian children and macaques (Bruce et al., 2018; Labo et al., 2019). EBV seropositivity was also found to be the strongest correlate of KSHV seropositivity in a rural Ugandan patient cohort (Sallah et al., 2020). Finally, EBV supports KSHV persistence after primary B cell infection and improves KSHV DNA maintenance after infection of EBV negative PEL *in vitro* (Bigi et al., 2018; Faure et al., 2019). Thus, KSHV might rely on EBV for its persistence, bidirectionally influencing their viral gene expression. I will primarily focus in this review on this interaction of EBV and KSHV in associated lymphomas.

## Contribution of Lytic γ-Herpesvirus Infection to Lymphomagenesis

One facet of how these two tumor viruses influence each other is that KSHV induces lytic EBV replication (McHugh et al., 2017). This is observed in double-infected B cells of humanized mice and double-infected PELs of patients. Surprisingly, this increased lytic EBV infection contributes also to the more frequent lymphomagenesis that is observed in KSHV and EBV infected humanized mice, because co-infection with lytic replication deficient EBV lacking the immediate early lytic activator BZLF1 (BamH1 Z fragment encoding leftward reading frame 1) does not cause more tumors than EBV single infection (McHugh et al., 2017). Similarly, BZLF1 deficient EBV infection was reported to cause fewer lymphomas than wild-type EBV infection in a smaller percentage of humanized mice (Ma et al., 2011; Antsiferova et al., 2014). This effect seemed more pronounced for lymphoma dissemination to liver and kidney than in spleen (Antsiferova et al., 2014). Vice versa, BZLF1 promotor variants that increase lytic EBV replication promote lymphomagenesis in humanized mice (Ma et al., 2012; Bristol et al., 2018). This was shown for a triple mutant (ZV, ZV’ and ZIIR) and the nasopharyngeal carcinoma associated V3 variant of the BZLF1 promotor. Decreased lymphomagenesis in the absence of lytic EBV replication is somewhat counterintuitive because infectious particle production is thought to lead to infected cell death, counteracting tumor cell proliferation.

Increased tumor formation in the presence of lytic EBV replication is, however, not only observed in humanized mice. Viruses that are enriched in EBV associated NK/T cell lymphomas and DLBCLs frequently carry deletions in the BART (BamH1 A fragment encoding rightward transcripts) miRNA encoding region that is thought to suppress lytic EBV replication by targeting expression of BZLF1 and the other lytic transactivator BRLF1 (Okuno et al., 2019). Moreover, plasma rather than cell-associated viral loads seems to be predictive of EBV associated tumorigenesis, such as nasopharyngeal carcinoma, PTLD, DLBCL, NK/T cell lymphoma and Hodgkin’s lymphoma, suggesting that lytic EBV replication is associated with these EBV associated malignancies (Kanakry et al., 2016).

**Figure 1:**
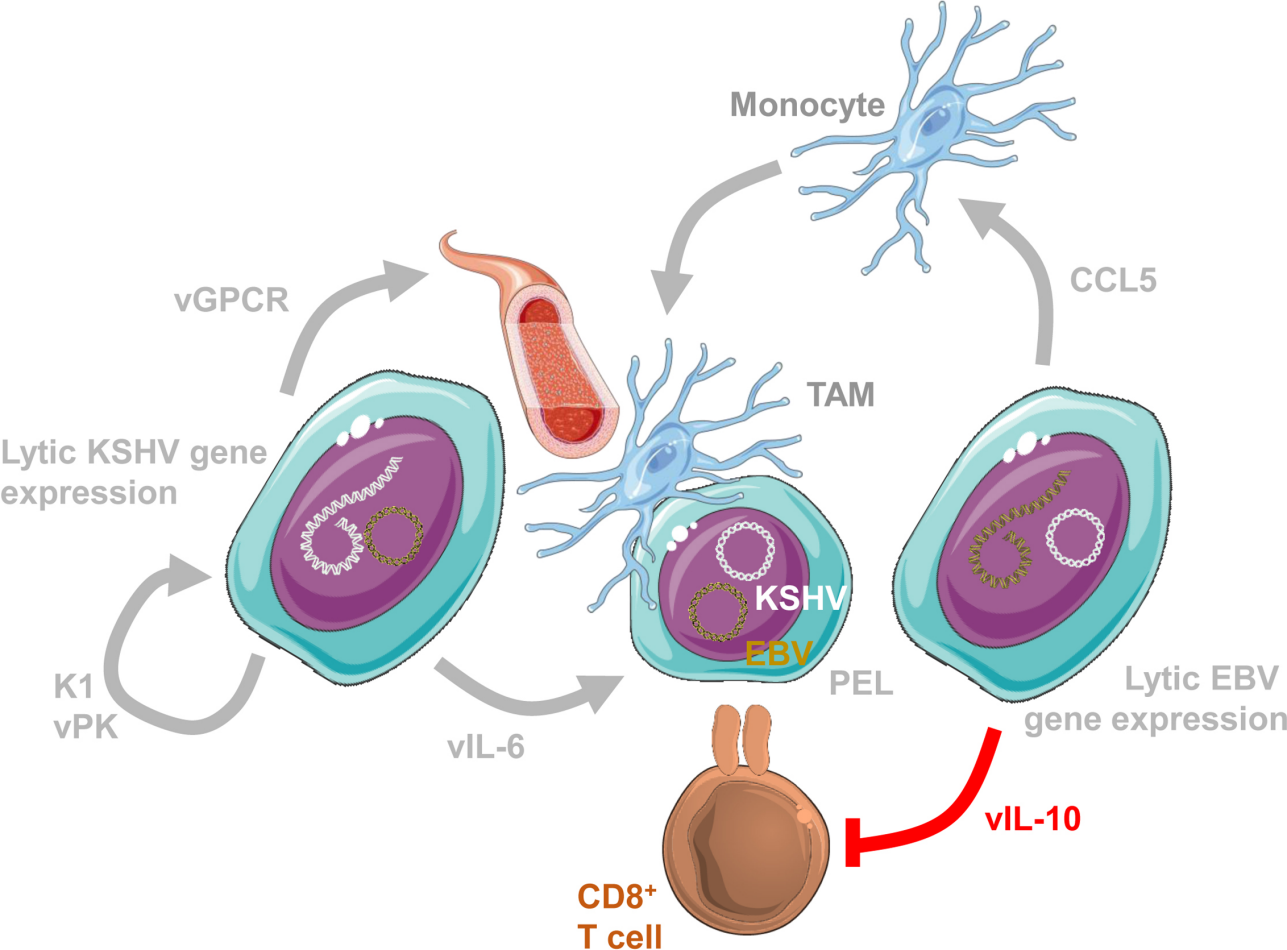
Lytic EBV and KSHV gene expression condition the tumor microenvironment. Conditioning of the tumor microenvironment by lytic EBV and KSHV gene products occurs most likely at the same time in primary effusion lymphomas (PELs) that are 100% KSHV and 90% EBV infected. Lytic EBV replication attracts monocytes *via* CCL5 to become immune suppressive tumor associated macrophages (TAMs). Viral IL-10 (vIL-10; BCRF1) suppresses CD8^+^ T cell recognition. In addition, the lytic KSHV product viral G-protein coupled receptor (vGPCR; ORF74) promotes angiogenesis. Furthermore, K1 (ORF-K1) and viral protein kinase (vPK; ORF36) promote proliferation of KSHV infected cells. Finally, viral IL-6 (vIL-6; ORF-K2) promotes KSHV infected B cell proliferation. This figure was created in part with modified Servier Medical Art templates, which are licensed under a Creative Commons Attribution 3.0 unported license: https://smart.servier.com.

How might such lytic EBV replication support tumor formation? It is likely that abortive early lytic EBV infection plays a pro-tumorigenic role (Münz, 2019). Accordingly, B cells transformed with a mutant EBV lacking the late lytic gene product BALF5 were more efficient in establishing lymphomas in immune compromised mice (Okuno et al., 2019). The pro-lymphomagenic effects of early lytic EBV gene products could be in part mediated by shaping the tumor microenvironment. Along these lines EBV transformed B cells with higher spontaneous lytic reactivation produce more tumour necrosis factor (TNF), CCL5 (RANTES) and IL-10 (Arvey et al., 2015) (**Figure 1**). In addition, EBV encodes also viral IL-10 (BCRF1) (Jochum et al., 2012). These could promote an immune suppressive tumor microenvironment through vIL-10 and IL-10 mediated T cell response suppression, as well as CCL5 dependent recruitment of myeloid suppressor cells (Casagrande et al., 2019; Walens et al., 2019). Similarly, early lytic KSHV gene products might promote lymphomagenesis (**Figure 1**). Along these lines viral IL-6 (ORF-K2) supports B cell lymphoma dissemination in immune compromised mice and B cell hyperproliferation in transgenic mice (Suthaus et al., 2012; Fullwood et al., 2018). Together with cMyc overexpression it can also support lymphoma formation in mice (Rosean et al., 2016). Thus, viral IL-6 probably serves as an auto- and paracrine growth factor for KSHV infected B cells. Furthermore, transgenic expression of viral protein kinase of KSHV (ORF36) in mice leads to B cell hyperproliferation and lymphoma development at increased frequency, compared to littermate mice (Anders et al., 2018). Viral protein kinase seems to facilitate B cell activation during KSHV infection. Moreover, also K1 transgenic mice develop lymphoproliferations and lymphomas in half of the animals (Prakash et al., 2000; Prakash et al., 2002; Prakash et al., 2005; Wang et al., 2007; Berkova et al., 2015). K1 encodes an activating receptor that promotes B cell stimulation and apoptosis resistance, including inhibition of Fas mediated extrinsic cell death induction. Finally, inducible expression of viral G protein-coupled receptor (ORF74) promotes angiogenesis and thereby Kaposi sarcoma-like tumorigenesis in mice (Yang et al., 2000; Holst et al., 2001; Guo et al., 2003; Montaner et al., 2003; Montaner et al., 2004; Jensen et al., 2005; Grisotto et al., 2006). Thus, early lytic gene products of both EBV and KSHV might condition the tumor microenvironment for more efficient malignancy development.

## Protection From Tumorigenesis by Lytic γ-Herpesvirus Replication Specific Immune Responses

In addition to this evidence from infections with mutant or variant γ-herpesviruses and early lytic viral gene product overexpression, protection by lytic replication specific immune responses against EBV and KSHV associated tumors points towards the importance of lytic infection in virus-associated malignancies. Uncontrolled lytic EBV replication might cause symptomatic primary EBV infection, called infectious mononucleosis (IM) (Luzuriaga and Sullivan, 2010). While two thirds of Europeans and Northern Americans acquire EBV prior to the age of two, the remaining one third often gets infected in the second decade of life (Dunmire et al., 2018). One third to half of these developed a strong CD8^+^ T cell lymphocytosis four to six weeks after EBV encounter. They shed infectious virus at increased titers into saliva often for months, develop antibodies against structural proteins like viral capsid antigen (VCA), but not yet EBNA1, and the majority of the expanding CD8^+^ T cells are directed against early lytic EBV antigens with individual peptide specificities constituting up to 40% of the total CD8^+^ T cell compartment (Callan et al., 1998). In contrast, latent EBV antigen specific CD8^+^ T cells emerge at IM convalescence and were therefore proposed to be the protective entity of EBV specific immune responses (Taylor et al., 2015). IM increases the risk to develop EBV associated Hodgkin’s lymphoma 4fold, but only for around 5 years after primary EBV infection (Hjalgrim et al., 2003). This was characterized in more than 50’000 adolescents and young adults in Scandinavian countries, as well as several follow-up studies (Hjalgrim et al., 2007; Hjalgrim et al., 2010). Therefore, uncontrolled lytic EBV replication could predispose for some EBV associated lymphomas.

Along with CD8^+^ T cells, natural killer (NK) cells expand during IM (Williams et al., 2005; Balfour et al., 2013; Azzi et al., 2014). Primarily, early differentiated NK cells expressing inhibitory NKG2A but not killer immunoglobulin-like receptors (KIRs) expand around 4fold (Azzi et al., 2014; Hendricks et al., 2014). These degranulate their cytotoxic molecules preferentially in the presence of lytically EBV replicating B cells (Chijioke et al., 2013; Azzi et al., 2014). Accordingly, NK cells of humanized mice which are also enriched in this early differentiated NK cell phenotype (Strowig et al., 2010) restrict wild-type but not BZLF1 deficient EBV infection (Chijioke et al., 2013; Landtwing et al., 2016). Restriction of lytic EBV replication by NK cells also reduced lymphoma formation, because NKp46 directed antibody depletion of NK cells leads to higher frequencies of DLBCL-like lymphomas in humanized mice (Chijioke et al., 2013; Landtwing et al., 2016).

Recognition of lytically EBV replicating B cells by NK cells seems to be primarily mediated *via* recognition by natural killer group 2 member D (NKG2D) with DNAX accessory molecule 1 (DNAM-1) as co-receptor (Pappworth et al., 2007). Their ligands are up-regulated on B cells upon induction of lytic EBV infection (Pappworth et al., 2007). NKG2D surface expression on NK and CD8^+^ T cells is reduced due to inefficient glycosylation by loss-of-function mutations in the Mg^2+^ transporter MAGT1 in patients with X-linked immunodeficiency with magnesium defect, Epstein-Barr virus (EBV) infection, and neoplasia (XMEN) (Chaigne-Delalande et al., 2013; Dhalla et al., 2015; Patiroglu et al., 2015; Brigida et al., 2017; Ravell et al., 2020a). EBV associated lymphomas develop in one third of the affected patients, but interestingly also one patient with Kaposi sarcoma was reported (Brigida et al., 2017; Ravell et al., 2020b). Mg^2+^ supplementation can restore NKG2D surface expression and EBV specific immune control in some of these patients (Chaigne-Delalande et al., 2013), but has not proven to be a successful durable therapy of XMEN (Ravell et al., 2020b). Nevertheless, NKG2D recognition of lytically replicating EBV infected B cells seems to be essential to prevent lymphomas.

In addition, the other primary immunodeficiencies that predispose for EBV associated diseases also seem to point towards cytotoxic lymphocytes, including NK and CD8^+^ T cells, as main components of EBV specific immune control (Damania and Münz, 2019; Latour and Fischer, 2019; Tangye and Latour, 2020). These affect the cytotoxic machinery (perforin, Munc13-4, Munc18-2), T cell receptor signaling (ITK, PI3K, RasGRP1, ZAP70, CORO1A), co-stimulation (CD27, CD70, 4-1BB, CTLA-4, SAP) as well as cytotoxic lymphocyte development and expansion (GATA2, MCM4, XIAP, STK4, CTPS1). While EBV specific immune control seems to be independent of type I and II interferons (IFNs) and antibodies (Latour and Fischer, 2019; Münz, 2020), type II IFN signaling seems to be required for KSHV specific immune control, and is compromised by mutations in IFNγR1 and STAT4 (Damania and Münz, 2019). Furthermore, the requirements for co-stimulation seem to be different with OX40 being essential for KSHV specific immune control (Byun et al., 2013). Nevertheless, T cells rather than B cells seem to be important for the immune control of both γ-herpesviruses.

Among these, adoptive transfer of early lytic EBV antigen specific CD8^+^ T cells has been shown to transiently control EBV infection in humanized mice (Antsiferova et al., 2014). Furthermore, late lytic EBV antigen specific CD4^+^ T cells have been demonstrated to control EBV transformed B cells in immune compromised mice (Linnerbauer et al., 2014). Both of these T cell specificities display cytotoxicity against EBV transformed B cell lines (Heller et al., 2006). Early lytic EBV antigen specific CD8^+^ T cell responses are also maintained at higher frequency than latent and late lytic antigen specific responses (Abbott et al., 2013). Similarly, KSHV lytic antigens are also more frequently recognized by CD4^+^ and CD8^+^ T cells (Robey et al., 2010; Roshan et al., 2017). Their protective functions against KSHV infected cells and in humanized mice need to be characterized in more detail in the future.

## Conclusions

Recent evidence suggests that most likely abortive early lytic replication in many cells or productive lytic replication in a few cells promotes KSHV and EBV associated lymphoma formation (Münz, 2019). In healthy virus carriers a large proportion of the cytotoxic CD8^+^ T cell response is dedicated to the recognition of early lytic KSHV and EBV antigens, probably more than to their latent antigens (Long et al., 2019). In contrast, most EBV specific vaccination approaches have so far focused on latent antigens to elicit protective T cells and late lytic antigens to induce antibodies (Taylor et al., 2004; Smith et al., 2006; Moutschen et al., 2007; Gurer et al., 2008; Ruiss et al., 2011; Meixlsperger et al., 2013; Kanekiyo et al., 2015; van Zyl et al., 2018; Rühl et al., 2019; Bu et al., 2019). From these studies the latent EBV antigens EBNA1, LMP1 and LMP2 have emerged as protective antigens (Rühl et al., 2020). In humanized mice, EBNA1 incorporated into an EBV derived virus-like particle (VLP), but not the VLP itself protected from challenge by EBV infection (van Zyl et al., 2018). In contrast to the B cell trophic VLP, EBNA1 targeting to dendritic cells (DCs) with recombinant antibodies and a potent adjuvant to activate classical DCs was not able to elicit sufficient T cell responses for protection (Gurer et al., 2008; Meixlsperger et al., 2013), even so both vaccines elicited primarily cytotoxic CD4^+^ T cell responses (Meixlsperger et al., 2013; van Zyl et al., 2018). Recombinant viral vectors are more efficient in eliciting CD8^+^ T cell responses and they can be combined with CD4^+^ T cell eliciting vaccines in heterologous protective vaccination (Rühl et al., 2019). For such comprehensively CD4^+^ and CD8^+^ T cell eliciting vaccines incorporation of early lytic EBV antigens, like BMLF1 (Antsiferova et al., 2014) should be considered. If proving efficient such vaccine formulations could then also be extended to lytic KSHV antigens. Thus, the new appreciation of a contribution of early lytic replication to possibly both EBV and KSHV associated tumorigenesis gives us also additional antigens that could be explored for vaccination against these two human tumor viruses.

## Author Contributions

The author confirms being the sole contributor of this work and has approved it for publication.

## Funding

Research in my laboratory is supported by Cancer Research Switzerland (KFS-4091-02-2017 and KFS-4962-02-2020), KFSP-Precision^MS^ and HMZ ImmunoTargET of the University of Zurich, the Cancer Research Center Zurich, the Baugarten Foundation, the Sobek Foundation, the Swiss Vaccine Research Institute, Roche, Novartis and the Swiss National Science Foundation (310030B_182827 and CRSII5_180323). The funders were not involved in the study design, collection, analysis, interpretation of data, the writing of this article or the decision to submit it for publication.

## Conflict of Interest

The author declares that the research was conducted in the absence of any commercial or financial relationships that could be construed as a potential conflict of interest.
